# Supplementary material for: Deep Learning-Assisted High-Throughput Analysis of Freeze-Fracture Replica Images Applied to Glutamate Receptors and Calcium Channels at Hippocampal Synapses
Source: Int J Mol Sci. 2020 Sep 14;21(18):6737. doi: 10.3390/ijms21186737 (PMC7555218; doi:10.3390/ijms21186737)
Supplement: Supplementary file 1 [file ijms-21-06737-s001.zip › Appendix A. Detailed Statistics_Kleindienst et al_proof.docx]

Appendix A. Detailed Statistics

All values represent mean ± SD.

**Figure 2:** **A**: Paired *t*-test followed by Holm–Sidak multiple comparison correction. SR vs. SO AMPAR: 117.5 ± 12.25 µm^−2^ (SR), 82.61 ± 17.9 µm^−2^ (SO), difference 34.9 ± 11.4, t_(3)_ = 6.096, *p* = 0.0177. SR vs. SO GluN1: 84.93 ± 22.83 µm^−2^ (SR), 96.63 ± 10.05 µm^−2^ (SO), difference 11.7 ± 29.6 t_(3)_ = 0.7901, *p* = 0.4872. **C**: 3-way ANOVA, within subject factors [receptor], [real vs. sim], [stratum]. [Receptor] F_(1,3)_ = 790.2 *p* < 0.0001; [real vs. sim] F_(1,3)_ = 7.017 *p* = 0.0771; [stratum] F_(1,3)_ = 0.0391, *p* = 0.856; [receptor] × [real vs. sim] F_(1,3)_ = 231.7, *p* = 0.0006; [receptor] × [stratum] F_(1,3)_ = 1.294, *p* = 0.338; [real vs. sim] × [stratum] F_(1,3)_ = 0.9732, *p* = 0.397, [receptor] × [real vs. sim] × [stratum] F_(1,3)_ = 2.053, *p* = 0.247. All comparisons that differ by only one factor were tested with Sidak post-hoc test, which showed significant differences for SR AMPAR vs. SR GluN1 (*p* = 0.0007), SO AMPAR vs. SO GluN1 (*p* = 0.0001), SR AMPAR vs. SR sim. AMPAR (*p* = 0.0291) and SO AMPAR vs. SO sim. AMPAR (*p* = 0.0059). **D**: Paired *t-*test followed by Holm–Sidak multiple comparison correction. SR: real vs. sim AMPAR t_(112)_ = 2.039, *p* = 0.126; real vs. sim GluN1 t_(109)_ = 0.838, *p* = 0.404. SO: real vs. sim AMPAR t_(75)_ = 4.121, *p* = 0.0004; real vs. sim GluN1 t_(106)_ = 1.955, *p* = 0.126.

**Figure 3:** **B**: Paired *t-*test followed by Holm–Sidak multiple comparison correction. SR: t_(118)_ = 4.371, *p* < 0.0001. SO: t_(91)_ = 3.89, *p* = 0.0002.

**Figure 4**: **A**: Paired *t-*test. SR vs. SO Cav2.1: 818.2 ± 187.5 µm^−2^ (SR), 772 ± 255.3 µm^−2^ (SO), difference 46.22 ± 136.1, t_(3)_ = 0.679, *p* = 0.5458. **D**: Paired *t-*test followed by Holm–Sidak multiple comparison correction. SR: t_(108)_ = 3.125, *p* = 0.0046. SO: t_(93)_ = 2.818, *p* = 0.0059. **E:** Paired *t-*test followed by Holm–Sidak multiple comparison correction. SR: t_(108)_ =2.616, *p* = 0.0116. SO: t_(94)_ = 2.823, *p* = 0.0116. **F:** Paired *t-*test followed by Holm–Sidak multiple comparison correction. SR: t_(108)_ = 3.677, *p* = 0.0004. SO: t_(93)_ = 3.942, *p* = 0.0003. **H:** Paired *t-*test. SR: t_(108)_ = 1.074, *p* = 0.285. SO: t_(93)_ = 1.329, *p* = 0.187.

**Figure S4: A**: Paired *t-*test followed by Holm–Sidak multiple comparison correction. SR vs. SO AMPAR: 66.68 ± 11.53 µm^−2^ (SR), 52 ± 7.35 µm^−2^ (SO), difference 14.67 ± 10.53, t_(3)_ = 2.786, *p* = 0.1325. SR vs. SO GluN1: 198 ± 22.05 µm^−2^ (SR), 191.7 ± 11.8 µm^−2^ (SO), difference 6.29 ± 30 t_(3)_ = 0.4186, *p* = 0.7037. **C**: 3-way ANOVA, within subject factors [receptor], [real vs. sim], [stratum]. [Receptor] F_(1,3)_ = 32.9, *p* = 0.0105; [real vs. sim] F_(1,3)_ = 3.639, *p* = 0.1525; [stratum] F_(1,3)_ = 2.088, *p* = 0.2442; [receptor] x [real vs. sim] F_(1,3)_ = 17.56, *p* = 0.0105; [receptor] x [stratum] F_(1,3)_ = 0.701, *p* = 0.4639; [real vs. sim] x [stratum] F_(1,3)_ = 0.0544, *p* = 0.8305, [receptor] x [real vs. sim] x [stratum] F_(1,3)_ = 4.361, *p* = 0.128. All comparisons that differ by only one factor were tested with Sidak post-hoc test, which showed significant differences for SR AMPAR vs. SR GluN1 (*p* = 0.0023), SO AMPAR vs. SO GluN1 (*p* = 0.0006) and SO AMPAR vs. SO sim. AMPAR (*p* = 0.0232) as well as a trend for SR AMPAR vs. SR sim. AMPAR (*p* = 0.0645). **D**: Paired *t-*test followed by Holm–Sidak multiple comparison correction. SR: real vs. sim AMPAR t_(82)_ = 0.846, *p* = 0.4; real vs. sim GluN1 t_(134)_ = 2.253, *p* = 0.076. SO: real vs. sim AMPAR t_(76)_ = 1.281, *p* = 0.367; real vs. sim GluN1 t_(148)_ = 3.313, *p* = 0.0046. **H**: Paired *t-*test followed by Holm–Sidak multiple comparison correction. SR: t_(112)_ = 6.4045, *p* < 0.0001, SO: t_(113)_ = 8.9507, *p* < 0.0001.

**Figure S5: C**: Paired *t-*test. 510.8 ± 102.5 (SR), 353.5 ± 33.46 (SO), difference 157.4 ± 75.52. t_(3)_ = 4.167, *p* = 0.0252. **D:** Paired *t-*test followed by Holm–Sidak multiple comparison correction. SR: t_(113)_ = 1.54, *p* = 0.126. SO: t_(115)_ = 3.232, *p* = 0.0032. **E**: 2-way ANOVA, within subject factors [stratum] and [real vs. sim]. [stratum] F_(1,3)_ = 0.1293, *p* = 0.7429; [real vs. sim] F_(1,3)_ = 1224, *p* < 0.0001; [stratum] × [real vs. sim] F_(1,3)_ = 2.975, *p* = 0.183. Sidak post-hoc test showed significant differences for SR real vs. SR sim. (*p* = 0.0173) and SO real vs. SO sim. (*p* = 0.0051).

**Figure S6:** **C**: Two-way ANOVA, within subject factors [stratum] and [real vs. sim]. [stratum] F_(1,3)_ = 0.0716, *p* = 0.8064, [real vs. sim] F_(1,3)_ = 0.2585, *p* = 0.6462, [stratum] × [real vs. sim] F_(1,3)_ = 0.2032, *p* = 0.6827.
