# Supplementary material for: Deep Learning-Assisted High-Throughput Analysis of Freeze-Fracture Replica Images Applied to Glutamate Receptors and Calcium Channels at Hippocampal Synapses
Source: Int J Mol Sci. 2020 Sep 14;21(18):6737. doi: 10.3390/ijms21186737 (PMC7555218; doi:10.3390/ijms21186737)
Supplement: Supplementary file 1 [file ijms-21-06737-s001.zip › Supplementary Figure captions_Kleindienst et al_proof.docx]

**Figure S1**. Examples of disagreements of manual and machine demarcation of PSD (**A**) or AZ (**B**). Scale bars: 100 nm.

**Figure S2.** Gold particle detection algorithm performance on single and double labelings. **A**: Example images showing unlabeled, as well as human, naïve Bayes and random forest detected 5 nm gold particles labeling Cav2.1 on active zones. 5 nm gold particles of this batch varied widely in size. Dashed red line indicates demarcated active zone area plus 30 nm outer rim. Gold particles were counted if their center lies within this line. **B**: Quantification of correct, false negative and false positive detection events by naïve Bayes and random forest algorithm. **C**: Fraction of wrong detection events for each image. This is calculated as $\frac{\#false positives+\#false negatives}{\#correct+\#false positives+\#false negative}$ for each image. Solid line indicates median, box indicates 1^st^ and 3^rd^ quartile, whiskers indicate 5^th^ and 95^th^ percentile and circles indicate outliers outside of 5^th^ or 95^th^ percentile. **D, E, F**: Same as above, but for double labelings of 6 nm gold particles (yellow) labeling AMPAR and 12 nm gold particles (red) labeling GluN1. Scale bars: 100 nm.

**Figure S3**. Comparison of different outer rim widths. Number of AMPAR particles (Mean ± SEM, n = 231 synapses) found in outer rims of different width. This graph is based on pooled data from SR and SO from the AMPAR labeling with 5 nm gold particles shown in Figure S5.

**Figure S4.** Glutamate receptor distribution in SR and SO PSD with switched gold particle sizes. **A**: Labeling density of AMPAR (12 nm) and GluN1 (6 nm). While AMPAR labeling density is higher in SR, the difference did not reach statistical significance (SR: 67 µm^-2^, SO: 52 µm^-2^, p = 0.13, n = 4 mice, paired t-test). GluN1 labeling density is similar in both strata (SR: 198 µm^-2^, SO: 191 µm^-2^, p = 0.7, n = 4 mice, paired t-test). Comparison with Fig. 2A indicates two-fold labeling efficiency of 6 nm gold-conjugated secondary antibody compared with 12 nm gold-conjugated one. **B**: Example image showing Monte-Carlo simulation of randomly distributed AMPAR particles. In this example image, the CPI for AMPAR and GluN1 is 1.1 and 0.45, respectively. Blue, red and orange circles indicate real AMPAR, real GluN1 and simulated GluN1 particles, respectively. Cyan overlay indicates demarcation of PSD, red dashed line indicates outer rim 30 nm from the demarcation. Scale bar: 100 nm. **C**: CPI of AMPAR and GluN1 particles in SR and SO. Dotted line at 0.548 indicates CPI of randomly distributed particles. Asterisks above the bars indicate significant difference from simulation. GluN1 particles are distributed similar to random distribution in both strata (SR: p = 0.96, SO: p = 0.41). AMPAR receptors in SO are distributed significantly more peripheral than expected by chance (p = 0.023) while there is a trend of peripheral preference in SR (p = 0.065). AMPAR particles are distributed more peripherally than GluN1 particles in both layers (SR: p = 0.0023, SO: p = 0.0006) (n = 4 animals, 3-way ANOVA with Sidak multiple comparison test; 266 partial and 35 complete PSDs were included in this analysis). **D**: Comparison of real and simulated NNDs between AMPAR or GluN1 particles. Real NNDs are slightly but significantly smaller than simulations for GluN1 in SO (p = 0.0046, n = 149 synapses) and show a trend of being smaller in SR (p = 0.076, n = 135 synapses). Real and simulated NNDs between AMPAR are similar in both strata (SR: p = 0.4, n = 83 synapses; SO: p = 0.37, n = 77 synapses) (paired t-test followed by Holm-Sidak multiple comparison correction). **E, F**: Significant positive correlation of AMPAR (**E**) or GluN1 (**F**) particle number with synapse size (AMPAR: SR: R^2^ = 0.3338, p < 0.0001, SO: R^2^ = 0.2223, p < 0.0001; GluN1: SR: R^2^ = 0.2181, p < 0.0001, SO: R^2^ = 0.3479, p < 0.0001, linear regression followed by Holm-Sidak multiple comparison correction). The regression line showed a trend of being steeper for GluN1 in SO than in SR (F = 3.231, p = 0.073). For AMPAR the regression lines in SR and SO were similar (F = 1.08, p = 0.3) (SR: n = 146 synapses, SO: n=155 synapses from 4 mice). This is at odds with our previous observation of significantly steeper slope in SR. The reason for this inconsistency could be that the lower labeling efficiency for AMPAR in this experiment makes it difficult to detect the difference in slope. **G**: Significant negative correlation between GluN1 and AMPAR density in both SR and SO (SR: R^2^ = 0.2699, p < 0.0001; SO: R^2^ = 0.1018, p = 0.0001, linear regression followed by Holm-Sidak multiple comparison correction). Regression line shows a trend of being steeper in SR (F = 3.774, p = 0.053) (SR: n = 146 synapses, SO: n = 155 synapses from 4 mice). **H**: NNDs between real AMPAR particles and real GluN1 particles are significantly larger than those between real AMPAR particles and simulated GluN1 particles in both layers (SR: p < 0.0001, n = 113 synapses; SO: p < 0.0001, n = 114 synapses), indicating subsynaptic segregation of AMPA and NMDA receptors (paired t-test followed by Holm-Sidak multiple comparison correction).

**Figure S5**. AMPAR distribution examined by single labeling with 5 nm gold particles. **A, B**: Example image of an SR (**A**) or SO (**B**) PSD. Blue and magenta circles indicate gold particle labeling for AMPAR and Monte-Carlo simulated AMPAR particles, respectively. The CPI for AMPAR in the synapse shown in **A** is 0.74, and the one in the synapse shown in **B** is 0.91. Cyan overlay indicates demarcation of PSD, red dashed line indicates outer rim 30 nm from the demarcation. Scale bars: 100 nm. **C**: AMPAR density is significantly higher in SR (p = 0.025, n = 4 animals, paired t-test). **D**: NNDs between real AMPAR particles are significantly smaller than NNDs between simulated AMPAR particles in SO (p = 0.0032, n = 116 synapses) but not SR (p = 0.13, n = 114 synapses) (paired t-test followed by Holm-Sidak multiple comparison correction). **E**: AMPAR particles are distributed significantly more peripherally than expected by chance in both layers (SR: p = 0.017; SO: p = 0.0051). Dotted line at 0.566 indicates CPI of simulated particles. CPI of AMPAR particles is similar in these layers (p = 0.96, n = 4 animals, 2-way ANOVA with Sidak multiple comparison test). **F**: AMPAR particle number is significantly correlated with PSD area in both layers (SR: p < 0.0001, n = 114 synapses; SO: p < 0.0001, n = 117 synapses). The regression line is significantly steeper in SR than SO (p < 0.0001, linear regression followed by Holm-Sidak multiple comparison correction).

**Figure S6**. Verification of AZ demarcation and CPI for Cav2.1. **A**: Example series of serial ultrathin section images through a synapse. Yellow overlay indicates the measured PSD length. Scale bar: 100 nm. **B**: Distribution of areas of complete AZs, demarcated with Darea software followed by manual correction, is not significantly different from that of PSDs, measured by serial section EM, in SR spine synapses (p = 0.6214, n = 38 AZs and 245 PSDs, Kolmogorov-Smirnov test). **C**: CPI of Cav2.1 particles in SR and SO is similar in both layers and not different from Monte-Carlo simulations (no significant main effect of either [Stratum] p = 0.81 or [Real vs Sim] p = 0.65 and no significant interaction p = 0.68, n = 4 mice, 2-way ANOVA, 142 partial and 62 complete AZs were included in this analysis). Dotted line at 0.539 indicates CPI of randomly distributed particles.
