# Supplementary figures and images for: Deep Learning-Assisted High-Throughput Analysis of Freeze-Fracture Replica Images Applied to Glutamate Receptors and Calcium Channels at Hippocampal Synapses
Source: Int J Mol Sci. 2020 Sep 14;21(18):6737. doi: 10.3390/ijms21186737 (PMC7555218; doi:10.3390/ijms21186737)

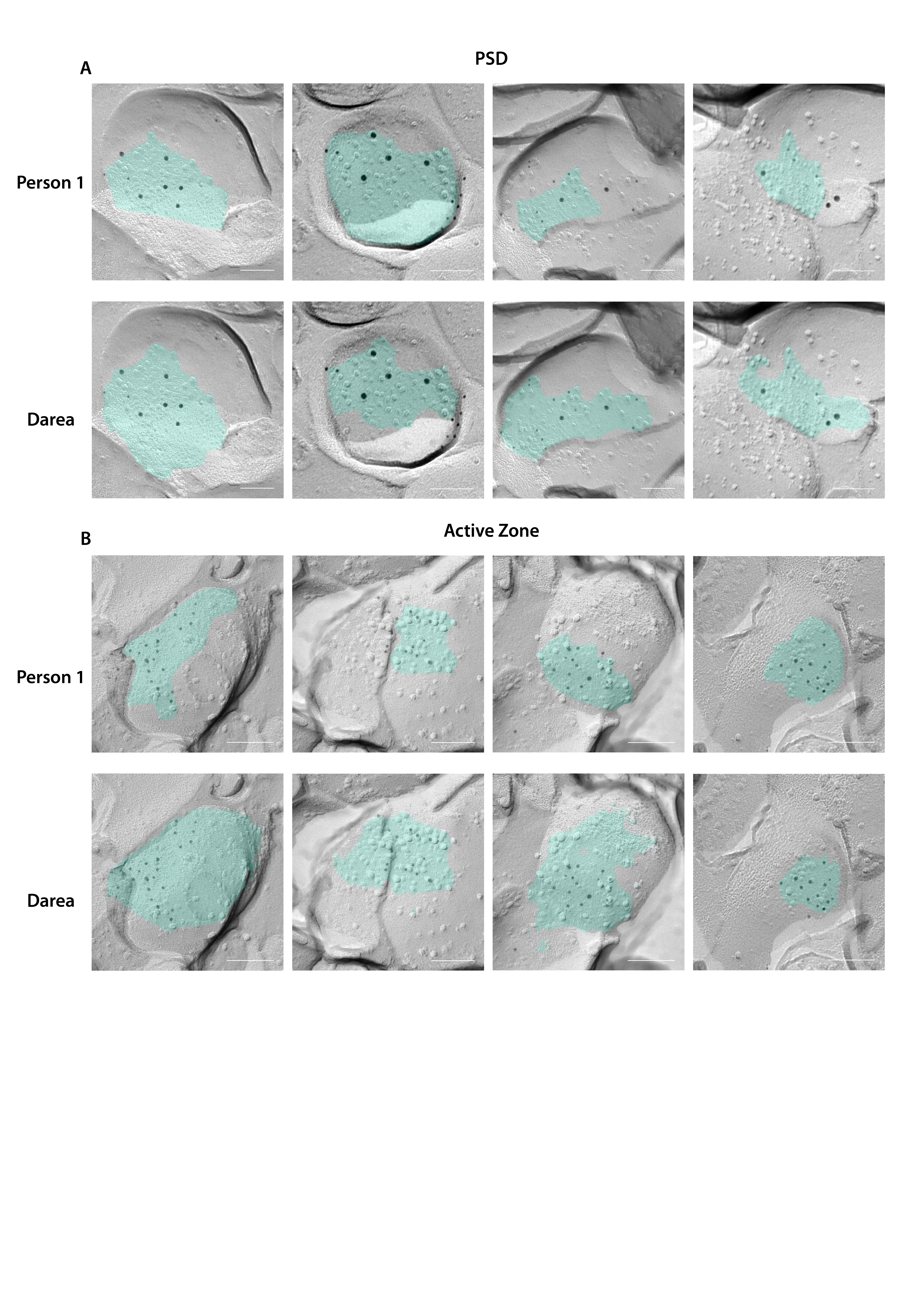

Supplement: Supplementary file 1 [file ijms-21-06737-s001.zip › Fig_Fig. S1.png]

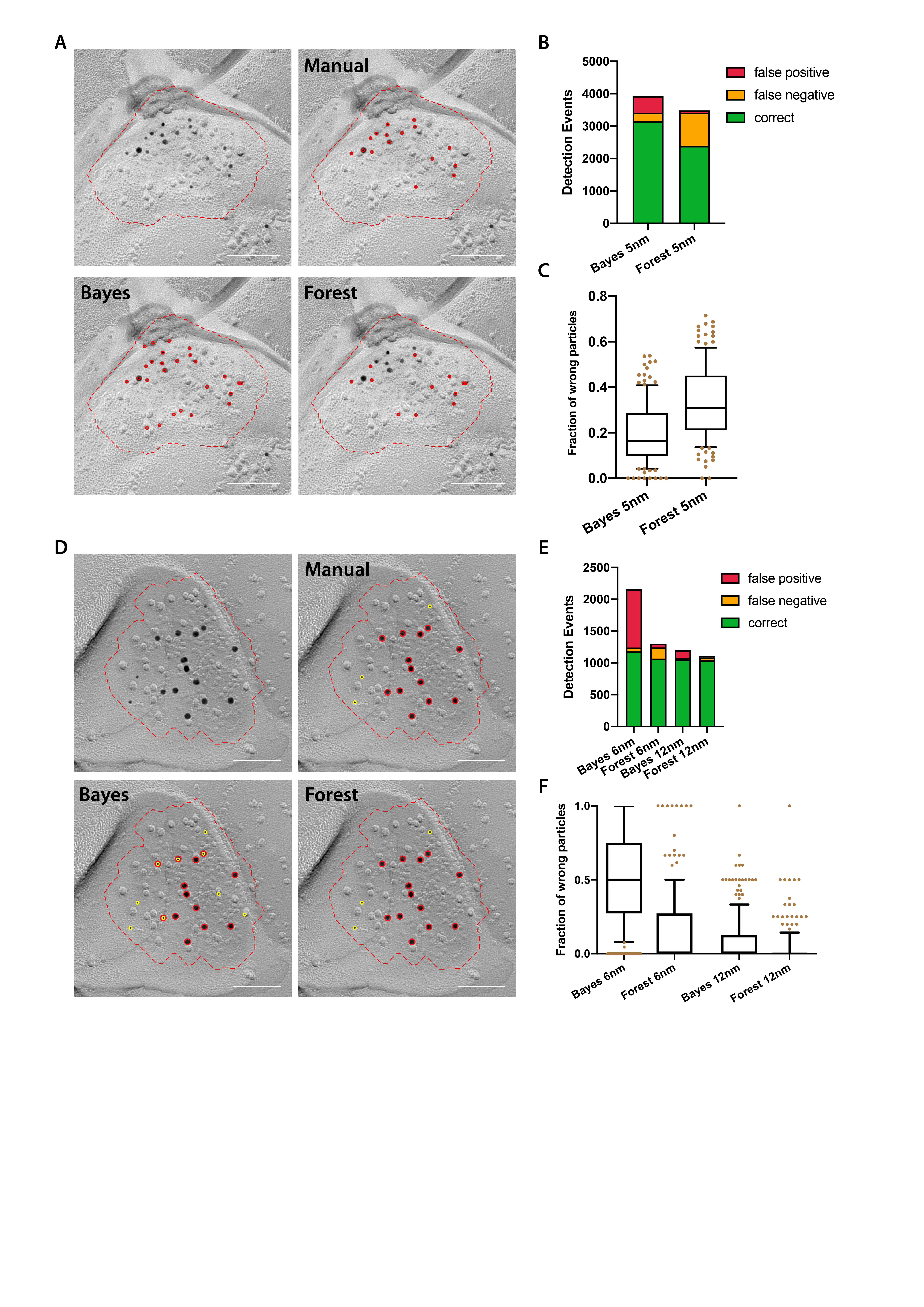

Supplement: Supplementary file 1 [file ijms-21-06737-s001.zip › Fig_Fig. S2.png]

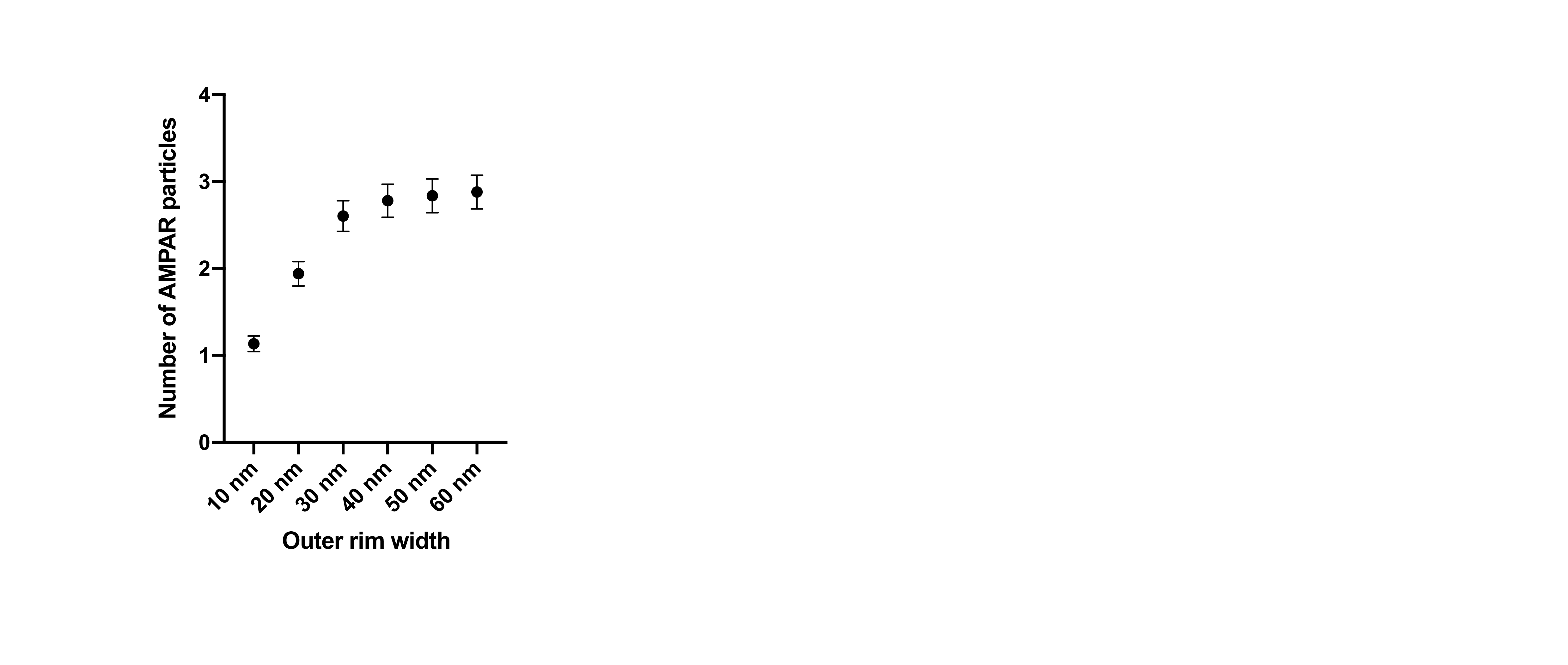

Supplement: Supplementary file 1 [file ijms-21-06737-s001.zip › Fig_Fig. S3.png]

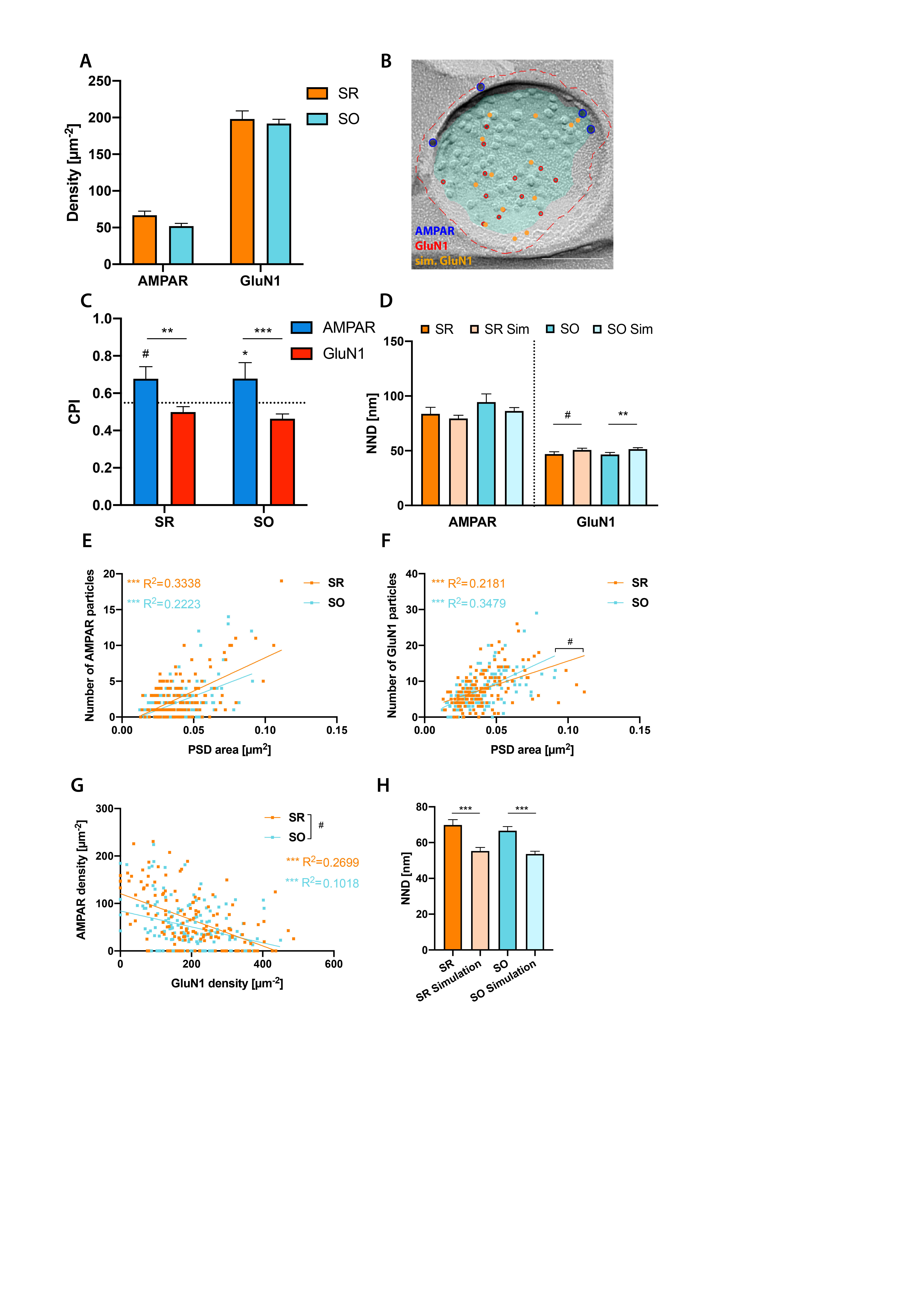

Supplement: Supplementary file 1 [file ijms-21-06737-s001.zip › Fig_Fig. S4.png]

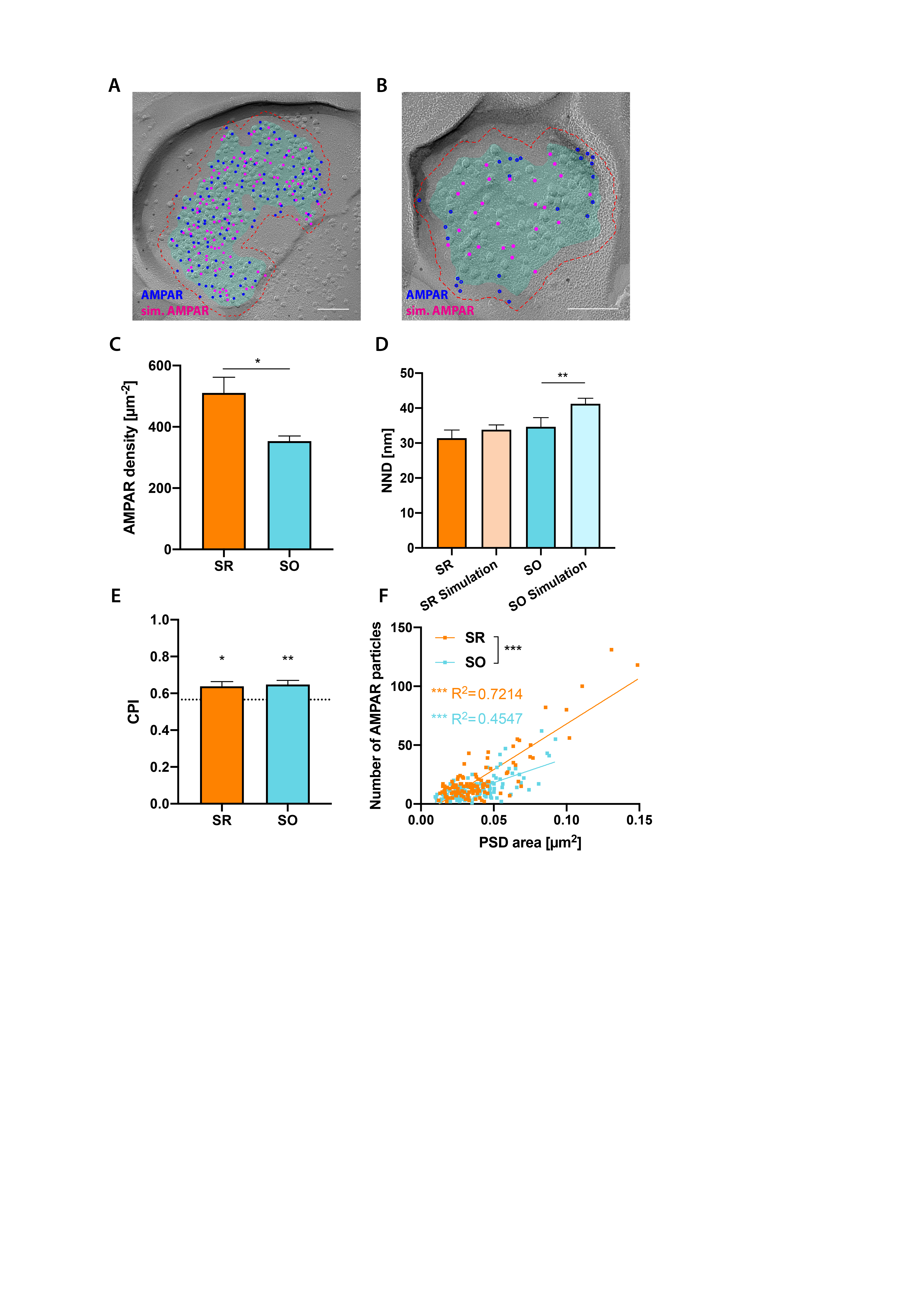

Supplement: Supplementary file 1 [file ijms-21-06737-s001.zip › Fig_Fig. S5.png]

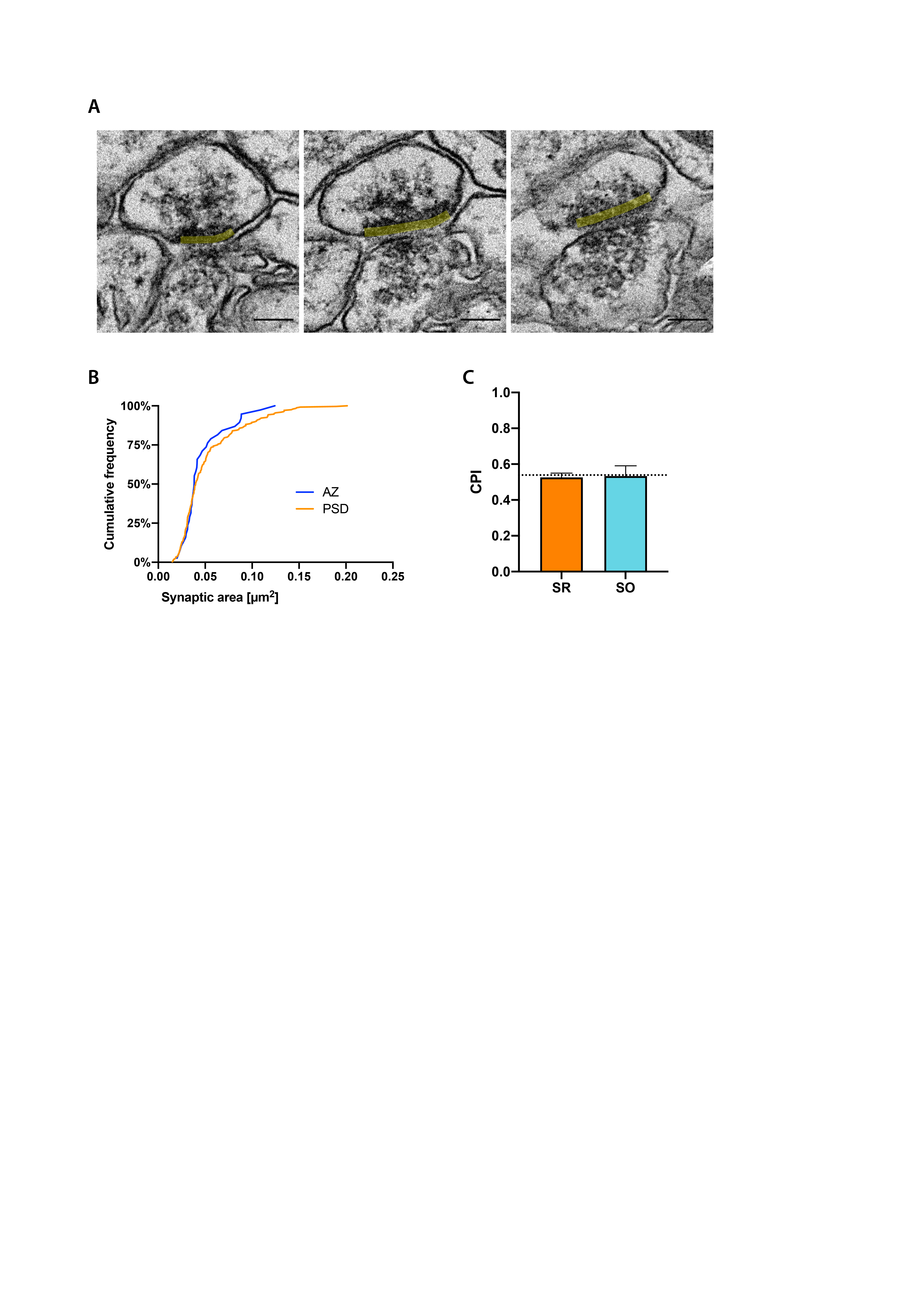

Supplement: Supplementary file 1 [file ijms-21-06737-s001.zip › Fig_Fig. S6.png]
